# Should We Be Screening for Ischaemic Heart Disease Earlier in Childhood?

**DOI:** 10.3390/children9070982

**Published:** 2022-06-30

**Authors:** Pier Paolo Bassareo, Stephen T. O’Brien, Esme Dunne, Sophie Duignan, Eliana Martino, Francesco Martino, Colin J. Mcmahon

**Affiliations:** 1 Mater Misercordiae Hospital, Mater, D07 R2WY Dublin, Ireland; 2 Children’s Health Ireland at Crumlin, D12 N512 Dublin, Ireland; stephen.obrien3@hse.ie (S.T.O.); duneees@tcd.ie (E.D.); sophie_duignan@hotmail.com (S.D.); cmcmahon992004@yahoo.com (C.J.M.); 3 School of Medicine, University College Dublin, Belfield, D04 V1W8 Dublin, Ireland; 4 Department of Paediatrics, La Sapienza University, 00185 Roma, Italy; elianamartino@inwind.it (E.M.); francesco.martino46@gmail.com (F.M.)

**Keywords:** atherosclerosis, childhood, coronary artery abnormalities, ischaemia, myocardial in farction, prevention

## Abstract

Ischaemic heart disease is the most common cause of death in males and the second in the female gender. Yet we often only focus on identification and treatment of this foremost cause of death in adulthood. The review asks the question what form of coronary disease do we encounter in childhood, what predisposing factors give rise to atherosclerosis and what strategies in childhood could we employ to detect and reduce atherosclerosis development in later life.

## 1. Introduction

Ischaemic heart disease is the most common cause of death in men and the second in women [1]. In a simplistic way, myocardial ischemia occurs when blood flow to the heart is reduced, preventing the cardiac muscle from getting enough oxygen. The reduced blood flow is usually the consequence of a partial or complete blockage of coronary arteries.

Although often considered as a condition affecting just adult people, life threatening myocardial ischaemia can present dramatically in paediatric age as well. This is the case of anomalous left coronary artery from the pulmonary artery (ALCAPA) and arterial calcification syndrome.

Not only that, but atherosclerosis—the most frequent aetiology of myocardial ischaemia—is a long process, which starts early since childhood in the form of fatty streaks. These first-stage lesions, which are characterised by an anomalous and reversible collection of oxidized lipoprotein particles in the inner layer of arteries, may lead to coronary arteries narrowing due to the progressive build-up of atheromatous plaque as time goes by. Fatty streaks are the first step of the atherosclerotic process [2]. Fatty streaks can be found in the aorta of a majority of children over the age of 3 years, augmenting quickly during adolescence, with coronary artery involvement which begins approx. a decade later [3]. Some characteristics of cardiovascular dysfunction during adulthood may even be influenced prenatally by means of a combination of genetic and unfavourable intrauterine environment [4].

Furthermore, type 2 diabetes is on an upward trajectory among children and adolescents. As per the report by the US Centers for Disease Control and Prevention (CDC), more than 5000 children and adolescents/year between the ages of 10 and 19, develop type 2 diabetes [5]. It is caused by insulin resistance as well as nonautoimmune β-cell failure usually starting amid puberty. However, youth-onset type 2 diabetes shares unique aspects, such as quicker β-cell decline and faster development of atherosclerosis and diabetes complications compared to the form developing in adulthood [6]. Microvascular complications such as endothelial dysfunction and risk factors for macrovascular complications are already evident at the time of diagnosis of type 2 diabetes in youth [7].

In addition, since the obesity epidemic is becoming a matter of serious concern worldwide, emerging countries included, this should be taken into account, since it represents an important risk factor for developing atherosclerosis. In fact, obesity onset in early life triggers atherosclerosis development in vessels such as the aorta and the coronary arteries [8]. There are also a couple of conditions in childhood which predispose to dramatically accelerated atherosclerosis, such as Kawasaki disease (KD) and familial hypercholesterolemia (FHC).

This review asks the question what forms of coronary disease do we encounter in childhood, what predisposing factors give rise to atherosclerosis and what strategies in childhood could we employ to detect and reduce atherosclerosis development in later life.

## 2. What Forms of Coronary Disease Do We Encounter in Childhood?

The forms of coronary disease seen in children can be subdivided into congenital and acquired. Those which are congenital can be symptomatic and life-threatening in paediatric age. Among them, the ALCAPA is worth mentioning.

ALCAPA is a well-recognised but rare congenital coronary artery anomaly affecting approximately 1 in 300,000 people [9]. Much less frequently encountered is the anomalous origin of either the right coronary artery or one of the conal branches off the pulmonary artery. ALCAPA typically presents in infancy resulting in myocardial ischemia and cardiac failure. The first reported case of a 3-month-old boy was by Bland, White and Garland in 1933 [10]. Other authors have reported several fatal ALCAPA, mostly fatal during the first year of life [11]. ALCAPA is usually an isolated entity, although it has been described in association with specific cardiac lesions (*e.g., ventricular septal defect, Tetralogy of Fallot and Hypoplastic left heart syndrome*) [12].

The pathophysiology of ALCAPA is intrinsically related to the decrease in pulmonary vascular resistance and pulmonary arterial pressure after birth. Therefore, the child often remains asymptomatic until a few weeks of age when the neonatal transitional circulation develops. With the drop in pulmonary arterial pressure, left coronary artery perfusion pressure decreases resulting in decreased antegrade coronary artery flow. The right coronary artery acts as the coronary blood supply as the pulmonary artery diastolic pressure drops further with reduced pulmonary vascular resistance, resulting in a coronary artery steal of the left coronary artery into the pulmonary artery. This induces myocardial ischemia conditioned by the extent of coronary arterial collateralisation, which impacts the timing of presentation [13].

**Figure 1:**
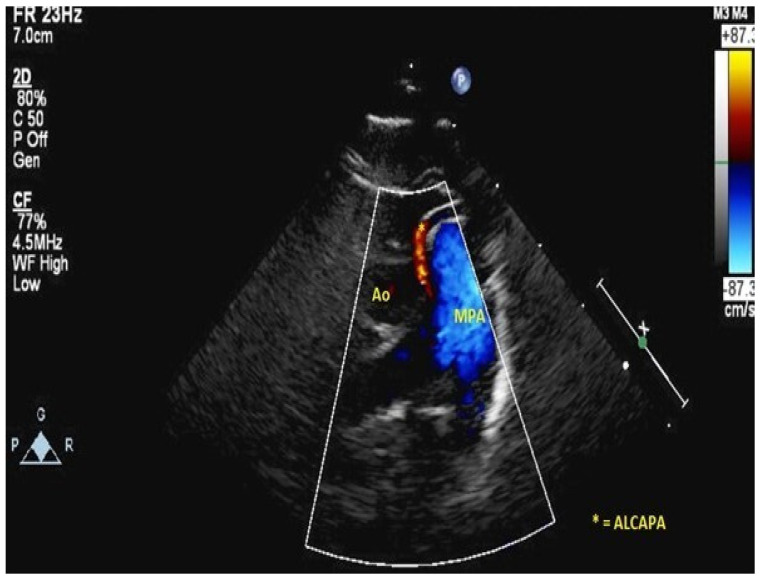
ALCAPA coming off the main pulmonary artery.

There are two types of presentation. Infants and small children typically have symptoms of ischemia during the first months of life (irritability, crying), poor feeding and congestive heart failure (left ventricular [LV] systolic dysfunction, LV dilatation, mitral regurgitation, enlarged right coronary artery with retrograde flow up the left coronary artery identified as a jet into the main pulmonary artery. See Figure 1).

Acute presentations such as sudden cardiac death are rare [14]. Acute clinical presentation may also be challenging to differentiate from myocarditis or dilated cardiomyopathy. The second type of presentation is in older patients with well collateralised coronary circulation who may present late in life and even be detected incidentally on postmortem examination.

Diagnosis depends on a combination of clinical findings, ECG abnormalities and imaging studies. The classic ECG presentation is Q waves in 1, aVL and leads V4–V6. Transthoracic echocardiography identifies an enlarged right coronary artery, retrograde flow in the left coronary artery into the pulmonary artery in combination with an ischemic mitral valve with mitral regurgitation and significantly dilated LV with reduced LV function [15]. Rarely, catheterisation or CT imaging is required to confirm the anatomical findings [16].

Treatment is surgical once the child is stabilised. Direct reimplantation of the coronary artery into the aorta is the preferable surgical procedure to ligation of the coronary artery [17,18]; although, in certain cases the creation of an intrapulmonary tunnel to baffle the coronary artery through the main pulmonary trunk to the aorta (Takeuchi procedure) is used [19]. Given its unique strategy, the Takeuchi procedures are associated with supravalvar pulmonary stenosis and baffle leaks [20]. A lot of patients demonstrate a dramatic clinical and echocardiographic improvement, with remodelling seen through reduction in LV size, improved LV function and reduction in mitral regurgitation [21]. Subsequent additional surgery such as mitral valve repair may be required in some patients and transplantation is rarely required after revascularisation [22,23,24].

Another symptomatic and usually deadly form of congenital ischaemic heart disease in infants is arterial calcification syndrome.

**Figure 2:**
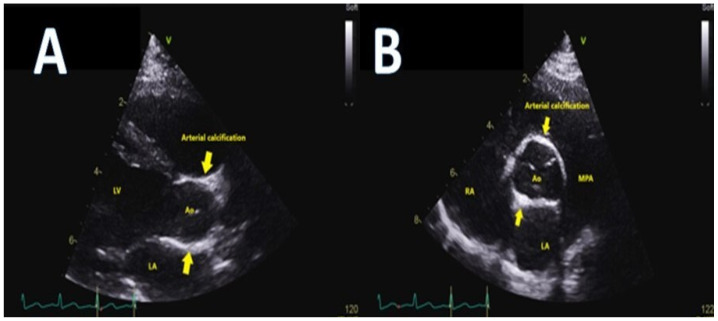
Thickening of the aorta patient seen in echocardiographic parasternal long axis (panel (**A**)) and short axis (panel (**B**)) views in arterial calcification syndrome.

Idiopathic calcification of the coronary arteries is an extremely uncommon inheritable disease that typically presents in early infancy [17]. Diffuse calcification of many of the arteries but particularly the coronary arteries results in cardiac ischemia, congestive cardiac failure and systemic hypertension [18,19]. The left and right coronary arteries are typically thick-walled, with significant luminal narrowing. The aorta, great vessels and renal vessels demonstrate thickening of the wall as well (Figure 2).

Histological examination demonstrates extensive accumulation of calcium in the inner elastic layer of all the vessels. Mutations in ENPP1 gene are linked with this rare syndrome in several patients [20].

Children typically present as very unwell, with congestive heart failure and arterial hypertension. Their combination should alert one to this diagnosis. Some children will present with angina-type symptoms and may even be inconsolable [21]. The pulses are often impalpable due to calcification, which can be visualised on plain X-rays of the arms and legs. Echocardiography is diagnostic demonstrating extensive aortic wall calcification and calcification of the coronary arteries [22]. Computed tomography may provide further delineation of the extent of coronary, aortic, great vessels and renal artery involvement [23]. Treatment consists of bisphosphonate therapy with some patients responding well to treatment [24,25]. Despite this, many patients die from hypertension, LV hypertrophy and myocardial infarction (MI) in the first three months of life, prompting some centres to offer cardiac transplantation [26,27,28,29,30,31,32,33,34,35].

Coronary artery disease in infants can also arise in subjects undergoing arterial switch operation—which implies coronary reimplantation—for transposition of the great vessels, a rare congenital heart disease. During surgery, the coronary arteries are translocated and coronary artery stenosis and blockage have been shown by angiography and computed tomography in up to 5–7% of subjects [36,37,38].

## 3. What Predisposing Factors Give Rise to Atherosclerosis and What Strategies in Childhood Could We Employ to Detect and Reduce Atherosclerosis Development in Later Life?

To summarise, there are two main acquired conditions predisposing to atherosclerosis development in children: Kawasaki disease (KD) and familial hypercholesterolemia (FHC).

KD is a systemic vasculitis with an unknown origin, maybe viral. It is the most common acquired cardiovascular disease in developed countries as well [39,40]. Although intravenous immunoglobulin infusion is an effective treatment for the disease, some patients still develop coronary aneurysms, which is an abnormal dilatation of the same [41,42]. Even if coronary artery aneurysm reversion is demonstrated in approx. 75% of patients, long-term outcomes of the inflamed arterial wall are uncertain, mostly in individuals with KD and giant aneurysms without regression [43]. These coronary aneurysms undergo remodelling with time, causing intimal (*the inner coronary artery layer*) thickening and calcification [44,45,46]. This triggers the onset of stenosis close to the aneurysms or blockage of the coronary arteries, causing ischemic heart disease [39,47]. Generally speaking, KD patients with previous small to medium coronary artery aneurysms show regression of the dilatation. Conversely, those with large coronary aneurysms (*e.g., those with a maximal internal coronary artery diameter ≥ 8.0 mm in terms of absolute dimension or those with a z-score ≥ 10 after correction for body size area, as per the American Heart Association classification*) are classified as having giant coronary aneurysms which do not revert, but continue or develop stenosis, causing acute MI easily [47,48]. In one of the largest studies on patients with KD and giant aneurysms and who were lost to follow-up for about 25 years, 46% of the sample exhibited a progression to coronary stenosis or complete obstruction. Of note 30.6% had MI and 15.4% died. 

The other patients had persistent coronary aneurysms without any noteworthy stenosis in the coronary artery over a follow-up between 10 and 25 years. 

Again, 21.7% of the patients showed obstructive coronary lesions that may trigger myocardial ischemia as time goes by. Similar findings are reported in smaller studies [43]. Ongoing remodelling of coronary arteries with giant aneurysms might continue long after acute KD, thus progressing to coronary stenosis, even 25 years following the occurrence of KD. The ongoing remodelling may be triggered by underlying ongoing inflammation of the coronary arterial wall, as shown by high blood levels of inflammatory markers in these patients [49]. In a 33 subject case series, abnormalities, such as arterial wall fibrosis, cellular infiltrates, hyperplasia of the intima or neovascularization, were found even in a few coronary artery portions without previous dilatation by echocardiography. Similar injuries were proved in significantly higher proportions in coronary tracts with long lasting aneurysms, followed by segments with angiographically regressed aneurysms and segments with coronary artery dilatation without any previous aneurysms [50]. 

**Figure 3:**
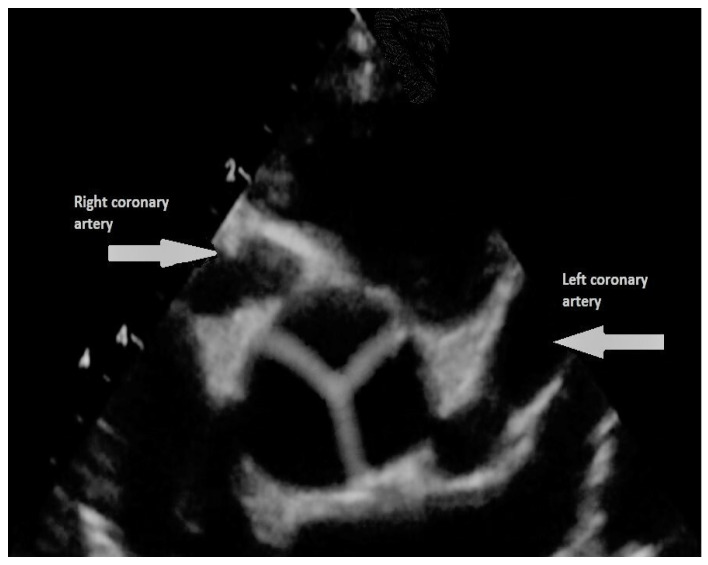
Persisting giant right and left coronary aneurysm (arrows) in a boy with previous Kawasaki syndrome (echocardiographic short-axis view).

The overall long-term survival of KD patients and giant aneurysms is quite good for up to three decades (survival rate is 90% at 20 years and 87% at 30 years) with various kinds of invasive interventions used in over 50% of the patients [43]. Adverse events are predicted by the extent of damage to the coronary arteries; patients with large coronary artery aneurysms are at high risk of complications. However, even those with remodelled aneurysms are not totally risk-free [44,49]. In accordance with the 2017 American Heart Association KD guidelines, long-term follow-up is mandatory for those with coronary artery involvement [51]. Patients with persisting large aneurysms require follow-up during their whole life with yearly or biyearly checks [51]. Blood thinning therapy in terms of primary prevention is suggested [51]. So far, no randomized clinical trials have been set up to evaluate the safety and efficacy of antithrombotic therapy to prevent coronary clots in KD. The current scientific evidence is based on small retrospective studies. Antiplatelet agents, such as low-dose aspirin, are considered standard of care to prevent clotting in individuals with coronary artery aneurysms. Patients with large or giant aneurysms are at very high risk of intracoronary thrombosis and MI (see Figure 3).

In aneurysmatic coronary arteries portions, clotting is induced by significantly abnormal slow blood flow and stasis. Because of that, patients are provided with a combination of antiplatelet and anticoagulant agents, usually low-dose aspirin and warfarin [51]. An important issue is when and how to detect myocardial ischaemia in patients long after the occurrence of the inflammatory disease. Even though it is suggested by the Guidelines as a useful tool to detect ischaemia during follow-up, a treadmill exercise stress test may not detect any ischemic alterations on ECG in patients with chest discomfort, while at cardiac catheterization a significant coronary artery stenosis is detected [51,52]. In agreement with this finding, a study suggested that the treadmill exercise stress test is the worst technique to detect myocardial ischemia in patients with KD [52]. Other possible modalities include stress echocardiography, stress magnetic resonance imaging and myocardial scintigraphy. They proved to be useful in terms of risk stratification, though some of them imply harmful radiations for such young patients [51]. Cardiac catheterization of the coronary arteries may be used for diagnosis and prognosis during the first year following KD and for recurrent follow-up every 1 to 5 years thereafter [51]. The reality is that it is still unknown how stopping the early progress of atherosclerosis in KD is complicated by coronary aneurysms.

Another recently manifested form of ischaemic heart disease in paediatric age is that triggered by COVID-19. The latter can infect children as well, thus causing the so-called Multisystem Inflammatory Syndrome in Children (MIS-C) also termed Paediatric Inflammatory Multisystem Syndrome (PIMS). MIS-C mimics some features of KD, toxic shock syndrome and macrophage activation syndrome. That is the reason why, at its first outbreak, some authors called it a Kawasaki-like disease. The relationship between MIS-C and COVID-19 infection suggests that the pathogenesis involves a post-infectious marked immune system dysregulation. MIS-C has an incidence of 0.2–0.6% of all paediatric COVID-19 infections [53,54,55]. Similarly to KD, MIS-C can also cause cardiac involvement with shock, myocardial dysfunction, ECG changes and coronary dilations/aneurysms development. The latter present in 15% of the MIS-C cases with cardiac involvement. The features of coronary aneurysms in MIS-C appear to be different compared with classic KD aneurysms. The most important differences are a lower likelihood of aneurysm formation than in classic KD, a reduced number of giant forms, a tendency towards aneurysm regression and less thrombotic events [56]. The long-term prognosis of this new form of coronary artery disease in children is still unknown, due to its too recent presentation.

Atherosclerosis is a lengthy chronic inflammatory and degenerative process, which begins in paediatric age and maybe even since foetal life, as shown in a number of studies [57,58]. It can be detected early as endothelial dysfunction, a preclinical abnormal reaction in which arteries constrict rather than dilating as a response to an appropriate trigger. Endothelial dysfunction is caused by reduced production and/or availability of nitric oxide and/or a mismatch in the relative contribution of endothelium-derived relaxing and contracting agents. It occurs before the development of atherosclerotic plaques. Endothelial dysfunction is involved in lesion development by promoting early and late pathways of atherosclerosis such as upregulation of adhesion molecules, increased chemokine production and leukocyte adhesion, raised cell permeability, enhanced low-density lipoprotein oxidation, platelet activation/adhesion/aggregation, cytokine release and arterial smooth muscle cell multiplication and migration [59].

**Figure 4:**
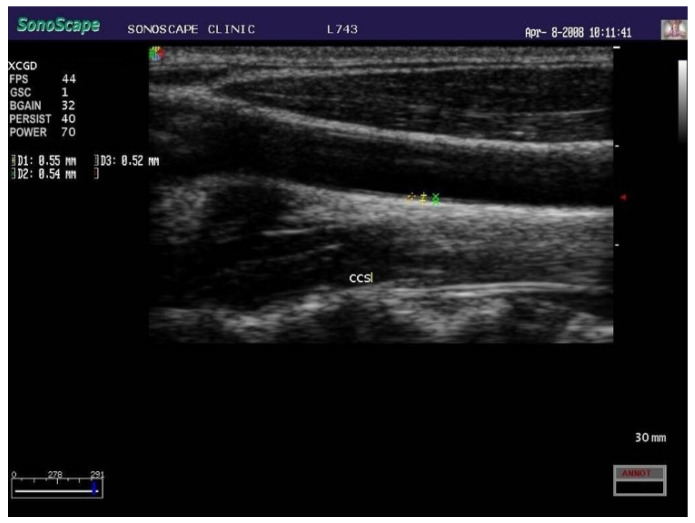
Carotid increased intima-media thickness in a child with FHC (ultrasound scan).

FHC exacerbates the above stated process. Raised inflammatory markers and early endothelial dysfunction have been demonstrated in children with FHC in association with increased intima-media thickness (Figure 4) and low-density lipoproteins (LDL) [60,61,62,63].

Coronary artery atherosclerotic plaques have been detected in 25% of children and adolescents with FHC and aged 11–23. Aortic atherosclerotic lesions can be found in a vast majority of adolescents with homozygous familial hypercholesterolemia (HoFHC) [64].

FHC is a genetic disorder with autosomal dominant transmission. FHC was the term coined by Carl Muller in 1938 to term the association of hypercholesterolemia, tendon xanthomas, xanthelasmas and occurrence of early ischaemic heart disease. Calcified aortic valve disease is not rare either [65,66]. LDL receptor (LDL-R) discovery by Goldstein and Brown in 1985 was the cornerstone to understand pathophysiology of LDL accumulation and atherosclerosis development [67,68,69]. Classic FHC (85–95% of cases) is caused by chromosome 19p13.2 mutations. It encodes for LDL-R. About 2000 possible mutations have been reported so far. LDL-R is a transmembrane glycoprotein that is responsible for degrading approx. two-thirds of circulating LDL. However, apolipoprotein B (~5–10%) as well as proprotein convertase subtilisin/kexin type 9 (PCSK9) (1–2%>) mutations can lead to a similar phenotype. More rarely, mutations can involve low density lipoprotein receptor adaptor protein 1 (LDLRAP1) (<1%, recessive FHC) or *apolipoprotein E* (APOE) (<<1%). Recessive FHC is diffused in some isolated geographic areas such as the island of Sardinia in Italy with a prevalence of 1:38,000 [70,71].

Regarding the prevalence of the disease, initially heterozygous familial hypercholesterolemia (HeFHC) was assumed to affect 1 in 500 individuals, while HoFHC prevalence was thought to be 1:1,000,000 [72]. The most recent epidemiologic investigations, however, have pointed out that the prevalence of HeFHC is 1:217–1:300, whereas that of HoFHC is 1:300,000. It means that HoFHC affects 30,000,000 individuals in the world [70,73,74].

The main feature of FHC is high LDL levels since infancy. Identifying children with FHC as early as possible is pivotal to start a treatment and improve their prognosis [75,76]. Unfortunately, missing FHC diagnosis is quite common in Europe with harmful aftermaths owing to the dramatically increased risk of MI after the age of 20 [75,77,78].

In adult patients, FHC diagnosis is made on the basis of increased LDL levels associated with normal triglycerides values, xanthomas, corneal arcus and family history of early MI [75,76].

Conversely, making diagnosis of FHC in childhood is by far more difficult. The only certain clinical feature is increased LDL. Xanthomas are rare. This is the reason why some different screening strategies to identify HeFHC have been suggested [79,80,81].

Over the last decade, many Guidelines and documents on FHC diagnosis and treatment have been published [75,82]. The criteria for suspecting FHC in paediatric age are summarised in Table 1 [83,84,85].

When FHC is suspected in a child aged 5 years or more, LDL should be checked. Secondary hypercholesterolemia should be ruled out as well [85]. Definite FHC diagnosis is based on genetic test. Making a diagnosis of FHC implies a cascade family screening as well [85,86].

The first therapeutic approach to FHC is based on lifestyle change (6–12 months), with family support. Calories and fat are important during children growth, but total fat intake should not go beyond 30% of overall calories. Saturated fat should not go beyond 7% of daily calories requirement [87].

Nutraceutical products have demonstrated the ability to reduce LDL in studies enrolling a small number of children. However, stanols and vegetable sterols are not suggested in children aged less than 6 years [88,89,90,91]. In children and adolescents with FHC, high viscosity glucomannan is capable of reducing LDL, blood pressure and weight [92,93]. Red yeast rice extract and policosanols decrease total cholesterol, LDL and apolipoprotein B in children affected by FHC [94].

Daily physical exercise (no less than 60 min/day) and no longer than 2 h/day spent on the TV, computer, smartphone, are important as well [95,96].

Lifestyle and diet change themselves are often inadequate in significantly reducing LDL levels in FHC children. As such, it means that taking medications is necessary.

Statins are the first line treatment in paediatric patients with FHC. The current Guidelines suggest that low dose statins should be provided in these subjects from the age of 8–10 years. The dose should be up titrated, when needed, to reach the goal of a 50% LDL reduction in comparison with baseline in children between 8 and 10 years or ≤3.5 mmol/L (130 mg/dL) in children aged ≥10 years, especially when additional risk factors, increased lipoprotein(a) included, are present [83]. In children with HoFHC, medical treatment should be started immediately after diagnosis [83].

In this setting, early statins administration is able to slow down atherosclerosis progression, as testified by measuring carotid intima-media thickness. The latter is capable of predicting major cardiovascular adverse events [97,98]. Statins have proved to be well tolerated in paediatric age so far, with rare side effects [99].

Ezetimibe, usually co-administered with statins, lowers LDL levels in FHC paediatric patients [100,101].

Monoclonal antibody therapy against PCSK9 (*e.g., evolocumab*) is indicated in HeFHC children and adolescents when lifestyle change and the highest tolerated dose of statins are not enough to normalise LDL. This treatment is usually efficient and well tolerated even in paediatric age [102].

More recently, other drugs (*lomitapide, mipomersen, bempedoic acid and anacetrapib*) acting through different mechanisms have been proposed in the most severe forms of FHC that are resistant to statins, but they have not been properly tested in children yet. In HoFHC children, lipoprotein apheresis to remove LDL and lipoprotein(a) from the blood is likely the best therapeutic option, though it is invasive and expensive [103].

Another poorly studied form of ischaemic heart disease is that manifested during neonatal asphyxia. Electrocardiogram changes with onset of T wave inversion and Q waves and cardiac enzymes fluctuations, troponin included, are similar to that observed in adults with heart attack. Echocardiography shows left ventricular dilatation and wall motion abnormalities. So far, the long-term clinical consequences of this neonatal myocardial infarction are still unknown [104].

## 4. Discussion

Ischaemic heart disease and atherosclerosis are often considered two conditions affecting just middle-aged subjects. On the contrary, they can occur and/or begin during childhood too. Ischaemic heart disease in paediatric age is congenital in its origin and it is usually a life-threatening condition in the affected infants. ALCAPA has a mortality rate up to 90% within the first year of life if left untreated. However, there are several cases reported in adolescents and adults. This is attributed to the development of collaterals between the right and left coronary arteries [105]. Without appropriate treatment, idiopathic infantile arterial calcification also has high morbidity and mortality, although studies to clarify them are still lacking, due to the rarity of the disease. On postmortem evaluation, calcifications of the large arteries are the main feature, but the most common gross findings are myocardial hypertrophy and stiff coronary arteries tortuosity [106]. These two conditions represent a real ischemic heart disease occurring in paediatric age.

On the other hand, fat accumulation and atherosclerosis are matters of concern from childhood. This is true not only in the general population, with paediatric obesity epidemic contributing to and exacerbating atherosclerosis through many pathways such as a rise in blood pressure and glucose level, anomalous lipid profiles and systemic inflammation, but mostly in KD and FHC patients. There is a strong link between LDL blood levels in children and the LDL levels in the same subjects during adulthood. A number of studies (*Bogalusa Heart Study, Muscatine Study, Cardiovascular Risk in Young Finns Study, Coronary Artery Risk Development in Young Adults [CARDIA]*) have shown that, since cholesterol tends to remain within the same percentiles throughout life, high cholesterol can be tracked from childhood to adulthood. Thus, children with higher cholesterol levels are more likely to become hypercholesterolemic adults. Family history of hypercholesterolemia is quite common as well and represents by far a significant risk factor for encountering major adverse cardiovascular events at a young age [107,108,109,110,111].

Childhood obesity is worrisome in terms of public health. A 2016 study estimated that 124 million children aged between five and nineteen were obese and nearly double that number overweight [112]. Landmark studies including the Bogalusa study showed a direct relationship between increasing body mass index and atherosclerotic lesions in prematurely deceased young people [113] Combined analysis from the International Childhood Cardiovascular Cohort identified obesity and overweight as positively correlating with high-carotid intima-media thickness, a noninvasive biomarker of structural atherosclerotic disease in young adults [114]. The pathophysiological basis of this relationship is multifactorial involving endothelial dysfunction, dyslipidaemia, chronic low-level inflammation and increasing arterial stiffness [115].

The aim of preventing or intervening on atherosclerosis development in childhood means methods for its earlier detection need to be developed. Serum biomarkers provide an exciting opportunity for enhanced, individualised risk prediction but progress in their use in the paediatric population for atherosclerosis has been slow. Targets that have been investigated include markers of oxidative stress, inflammatory markers, lipoproteins and endothelial adhesion molecules [116]. Noninvasive imaging techniques using high resolution ultrasound such as carotid and aortic intimal-medial thickness are validated to assess early atherosclerotic changes. Children with hypercholesterolaemia have increased carotid intimal thickness compared to those with normal cholesterol levels [117]. It has the benefit of offering direct information about the individual’s vascular health, but further longitudinal studies are required to assess its usefulness in the prediction of adult cardiovascular disease in paediatric patients [118]. Metabolomics, the most promising of omics sciences, which is able to take a picture of the metabolic state of an individual in physiological as well pathological settings, may represent an important tool to unveil a number of still obscure points [119].

## 5. Conclusions

While some strategies have been developed to identify those people at increased risk of atherosclerosis-related cardiac ischaemia and MI, and to treat them in terms of primary prevention, the lack of robust data on the effectiveness of the latter is still a weakness and much more research in the field will be needed in the foreseeable future.

## Figures and Tables

**Table 1:** Criteria suggesting high probability of FHC in paediatric patients [83,84,85].

Criterion	Title 2
1	Occasional finding of LDL ≥ 4 mmol/L (160 mg/dL) without any bodily sign of note and with a parent suffering from hypercholesterolemia or ischaemic heart disease (<55 years in males and <60 years in females)
2	LDL-C ≥ 5.0 mmol/L (190 mg/dL) in two different checks after 3 months of low cholesterol diet
3	LDL-C ≥ 3.5 mmol/L (130 mg/dL) and one parent with a FHC genetic diagnosis
4	LDL-C ≥ 13 mmol/L (500 mg/dL) with cutaneous xanthomas (dominant or recessive HoFHC)

## Data Availability

Not applicable.

## Abbreviations

ALCAPA	anomalous left coronary artery from the pulmonary artery
KD	Kawasaki disease
FHC	familial hypercholesterolemia
LV	left ventricular
ECG	electrocardiogram
Echo	echocardiogram
CT	computed tomography
MI	myocardial infarction
LDL	low density lipoprotein
HoFHC	homozygous family hypercholesterolemia
HeFHC	heterozygous hypercholesterolemia
LDL-R	low density lipoprotein receptor
PCSK9	proprotein convertase subtilisin/kexin type 9
